# Publication rates of pharmacy residents involved in a team-based research program

**DOI:** 10.1093/ajhp/zxac233

**Published:** 2022-08-18

**Authors:** Kiya K Bennett, Ashley N Fox, Jamie L Miller, Stephen Neely, Vincent C Dennis, Peter N Johnson

**Affiliations:** University of Oklahoma College of Pharmacy, Oklahoma City, OK, USA; University of New Mexico Health System, Albuquerque, NM, USA; University of Oklahoma College of Pharmacy, Oklahoma City, OK, USA; University of Oklahoma College of Pharmacy, Oklahoma City, OK, USA; University of Oklahoma College of Pharmacy, Oklahoma City, OK, USA; University of Oklahoma College of Pharmacy, Oklahoma City, OK, USA

**Keywords:** pharmacy residency, publication rates, research, team-based research program

## Abstract

**Purpose:**

The University of Oklahoma College of Pharmacy (OUCOP) implemented an individualized residency research committee and skill development program to facilitate completion and publication of research projects. The purpose of this study was to evaluate the outcomes the program had on project publication rates and subsequent publications after graduation for postgraduate year 1 (PGY1) and postgraduate year 2 (PGY2) residents.

**Methods:**

This study included OUCOP PGY1 and PGY2 residents from classes graduating from 2011 through 2019. Literature searches for all resident projects and subsequent publications were performed. Data collection included residency type (PGY1 vs PGY2), initial position after residency, and project type. The primary objective was to identify the publication rate of research projects. Secondary objectives included a comparison of the number of publications after residency graduation between residents who did and did not publish their residency project and analysis of factors associated with subsequent publications. Zero-inflated Poisson regression was utilized to analyze subsequent publication status controlling for other factors. Statistical analyses were performed using SAS/STAT with an a priori *P* value of <0.05.

**Results:**

Eighty-two projects were completed by 73 residents. Forty-three of 82 projects were published (52.4%) by 39 of 73 residents (52.1%). After residency graduation, 54 residents (74.0%) had a subsequent publication. Factors associated with subsequent publications were initial position in an academic role and completion of additional training after residency.

**Conclusion:**

After implementation of the program, the majority of residents published their projects and had subsequent publications. Future efforts should be taken to identify opportunities to foster independence in research and scholarship for residents.

**Key Points**

- A team-based research program was initiated to facilitate completion of residency research projects and increase the likelihood of publication.
- The overall publication rate following implementation of this program was 52%, although publication rates varied from 25% to 88% depending on the postgraduate year 1 or 2 program.
- The majority of residents had subsequent publications, and there were higher odds of subsequent publication after residency graduation among those who took an academic position or completed additional training (eg, a fellowship or graduate school) after residency vs those who took a position as a clinical pharmacist.

As a mechanism to develop research skills in pharmacy residents, the American Society of Health-System Pharmacists (ASHP) requires that accredited programs provide postgraduate year 1 (PGY1) and postgraduate year 2 (PGY2) pharmacy residents the opportunity to conduct a practice-based research and/or quality improvement project and to prepare a manuscript suitable for publication.^1-3^ Participation in a project allows residents to develop essential skills, such as critical thinking, collaboration with other healthcare providers, and development of a structured process to conduct their own projects.^4^ Further, these projects have the potential to impact the institution in which they are conducted and, if published, the health system at large. However, the publication rates of residency projects have historically been variable, ranging from 2% to 86%.^5-12^

Previous research has focused on identifying publication barriers for residency projects. Barriers include lack of mentorship, inadequate protected time, and lack of previous research experience.^5,6,13^ Currently, there are a paucity of studies evaluating individualized research committees coupled with structured research skill development and the impact this may have on resident project publication rates. It is essential to understand factors affecting resident publication rates and seek to improve mentorship, expand knowledge of research, and teach critical thinking and time management skills for conducting a research project.^4^ The purpose of this study was to evaluate the outcomes of an individualized research committee and research skill development program at the University of Oklahoma College of Pharmacy (OUCOP) on resident project publication rates and subsequent publications after graduation. The rationale for evaluating these 2 variables was to explore whether providing residents with a structured research experience including foundational topics and mentors impacts publication of completed research projects as well as subsequent future publications in practice.

## Description of individualized research committee and research skill development program

Before the graduating class of 2011, the OUCOP residency oversight committee, the Residency Review Committee (RRC), noted that many residents had difficulty completing their research projects and thus were unable to submit their manuscripts for publication. As a result, the RRC implemented a policy involving individualized research committees to provide mentorship, support, and feedback to PGY1 and PGY2 pharmacy residents on the development and completion of projects beginning with the residency class graduating in 2011. A research committee is developed for each resident and consists of a primary content mentor, a residency program director (RPD), at least one additional content and/or practice expert (eg, a clinical pharmacist and/or physician), and a faculty or staff member with expertise in study design and/or biostatistics (eTable 1). In May of each academic year, the RRC solicits potential project topics from residency preceptors who are interested in serving as content mentors. The content mentors identify topics that have not been fully developed and can feasibly be completed within 1 year. The RRC reviews this potential list of topics to evaluate feasibility and then distributes a final list of the proposed projects for RPDs to share with incoming residents during May of each academic year. Incoming residents work with their RPDs to select a research project by mid-June. Once a resident selects a topic, the RPD works with the content mentor to develop the research committee, with the goal of finalizing the committee by the end of June of each academic year. In situations where a content mentor is less experienced with research, the RPD works with the RRC to ensure that at least one committee member has more research experience.

A description of project timelines and duties for the resident, RPD, content mentor, and committee is provided in eTable 1. The primary content mentor guides the resident in every phase of the research project and provides formative and summative feedback longitudinally through PharmAcademic. Research rotations are conducted over 12 months; however, beginning in 2010, our residency programs began offering a required 1-month concentrated research rotation for dedicated data collection. During quarterly preceptor meetings, content mentors provide an overview of their resident’s performance and progress. The RPDs provide updates to the RRC on each resident’s project.

This program also includes structured research skill development to introduce residents to the research process and develop skills needed to successfully complete and submit a project for publication. This is implemented through several required educational research discussions ranging from 7.5 to 12 hours in length and covering a variety of topics, including those focused on the institutional review board (IRB) and interactive sessions focusing on working with the research committee, medical writing, and biostatistics (eTable 1).^14,15^ These interactive sessions are strategically organized to coincide with the timeline of research activities and are facilitated by faculty and staff with expertise in biostatistics and research design and extensive experience in scholarship and research.

## Methods

This IRB-approved study included PGY1 and PGY2 residents from July 1, 2010, to June 30, 2019. Residents were identified by graduation records maintained by the RRC containing the resident’s project title and their primary content mentor. The 2019 end date was selected to allow sufficient time for publication, as previous studies have noted that the mean time to publication of residency projects is approximately 2 years.^8,9^ Residency graduation records were utilized to identify initial position after graduation and the theme of the research project.

### Data collection and study objectives

The publication status of each resident project was determined and confirmed by 2 investigators performing systematic searches in MEDLINE, EMBASE, Google Scholar, and Scopus through June 30, 2021. Search terms included the resident’s first and last names and relevant key terms from the project title. Searches that returned no citations or citations determined to be unrelated to the resident of interest were repeated using the name of the respective research content mentor. If no citations were found, the project was categorized as unpublished.

Data collected for published projects included the full journal citation, time from residency graduation to publication date, number of authors on the publication, author order of the resident, journal type (ie, medical or pharmacy), and impact factor of the journal (obtained for the year of publication, if available). In addition, the highest H-index score for the resident’s coauthors was identified using the Scopus database. The H-index is utilized to measure a researcher’s contribution to the literature, including the significance and overall impact of their research.^8,16^ For all residents, whether their residency project was published or not, a second literature search was conducted by 2 investigators to identify subsequent publications after residency graduation. The number of publications was collected for each year starting from January in the first year after residency graduation until June 30, 2021.

The primary objective was to identify the publication rate of residency research projects. Secondary objectives included characterization of the publications based on journal type and impact factor and a comparison of publication quantity after residency graduation among residents who did and did not publish their residency project. Additional secondary objectives included an analysis of factors associated with time to publication of the residency project and subsequent publications after residency graduation.

### Statistical analysis

Data were collected and managed using REDCap (Research Electronic Data Capture) electronic data capture tools.^17,18^ Comparisons of continuous data between those who published their residency project vs those who did not were made using Student’s *t* tests and Wilcoxon statistics, as appropriate. Comparisons of categorical data between groups were conducted with *χ*^2^ or Fisher’s exact tests, as appropriate.

Stepwise Cox regression was used to analyze time to publication of the residency project. A random effect was added to the model to account for clustering of residents who completed more than one residency at OUCOP. If the project was not published, it was considered a censored observation and the elapsed time from graduation to June 30, 2021, was used. Residency type, graduation year, and first position after residency were considered for inclusion and were entered into the model if the initial *P* value was less than 0.25 and remained if the *P* value was less than 0.15. Adjusted hazard ratios (aHRs) and 95% confidence intervals (CIs) were reported as well as the probability of publishing more quickly than the reference group. Zero-inflated Poisson (ZIP) regression was used to analyze subsequent publication status after residency graduation while controlling for other factors, owing to the excess of residents who had no publications. ZIP regression simultaneously generated logit (subsequent publications vs no publications) and Poisson (number of subsequent publications) models. Statistical analyses were performed using SAS/STAT software, Version 9.4 (SAS Institute, Inc., Cary, NC, USA), with an a priori *P* value of <0.05.

## Results

A total of 82 projects completed by 73 residents were included. Ten residents (13.7%) completed both their PGY1 and PGY2 residencies with OUCOP. Of the 82 projects, 27 (32.9%) were completed by PGY1 residents, including PGY1 community (n = 9; 11.0%), PGY1 managed care (n = 1; 1.2%), and PGY1 pharmacy (n = 17; 20.7%) residents. Fifty-five projects (67.1%) were completed by PGY2 residents, including in ambulatory care (n = 21; 25.3%), cardiology (n = 6; 7.2%), human immunodeficiency virus (HIV) pharmacy (n = 8; 9.6%), internal medicine (n = 8; 9.6%), oncology (n = 4; 4.8%), and pediatric (n = 8; 9.8%). The majority of these projects (n = 67; 80.7%) were retrospective. Fourteen (16.9%) were prospective, and 2 (2.4%) had a prospective and retrospective component. The theme for the majority of projects involved a clinical focus (n = 57; 68.7%); however, other themes included behavior (n = 12; 14.5%), pharmacoeconomics (n = 2; 2.4%), quality improvement/pharmacy operations (n = 11; 13.2%), and scholarship of teaching and learning (n = 1; 1.2%).

Overall, 43 projects included in the study (52.4%) were published. The publication rate differed among graduation years from 20.0% to 72.7%, but no statistically significant difference was noted over the 9-year period (*P* = 0.33) (8/11 [72.7%] in 2011, 6/9 [66.7%] in 2012, 5/11 [45.5%] in 2013, 5/11 [45.5%] in 2014, 7/10 [70.0%] in 2015, 5/8 [62.5%] in 2016, 4/9 [44.4%] in 2017, 1/5 [20.0%] in 2018, 2/8 [25.0%] in 2019). Of the 73 residents, 38 (52.1%) published at least one project. Of the 10 who completed both PGY1 and PGY2 residencies at OUCOP, 4 published both projects, 1 published only their PGY1 project, and 4 did not publish either project. One additional resident’s project spanned both their PGY1 and PGY2 residencies and was published following the PGY2 residency year.

Table 1 provides a comparison of variables among residents who did and did not publish their projects. There was no statistically significant difference in first position after graduation, with the majority in both groups taking a clinical pharmacist position. There was also no difference in study design or PGY1 vs PGY2 status between the groups, but there was a significant difference in the theme of the project between the groups (*P* = 0.02).

**Table 1.** Comparison of Variables in Residents Who Published Their Research Projects vs Those Who Did Not (N = 82)^a^

Variable	Unpublished research	Published research	
Results by resident (n = 73)	n = 35	n = 38	*P* value
First position after residency			0.14
Clinical pharmacist	26 (74.3)	22 (57.9)	
Academia	9 (25.7)	13 (34.2)	
Additional training (graduate school, fellowship)	0 (0)	3 (7.9)	
Results by project (n = 82)	n = 39	n = 43	
Residency type			0.59
PGY1	14 (35.9)	13 (30.2)	
PGY2	25 (64.1)	30 (69.8)	
Study design of project			1.0
Prospective	7 (18.0)	7 (16.3)	
Retrospective	31 (79.5)	35 (81.4)	
Retrospective and prospective	1 (2.6)	1 (2.3)	
Theme of project			0.02
Behavioral	6 (15.4)	6 (14.0)	
Clinical	22 (56.4)	34 (79.1)	
Pharmacoeconomics	2 (5.1)	0 (0)	
Quality improvement/pharmacy operations	9 (23.1)	2 (4.7)	
Scholarship of teaching and learning	0 (0)	1 (2.3)	

Abbreviations: PGY1, postgraduate year 1; PGY2, postgraduate year 2.

^a^Data shown as No. (%).

Table 2 provides a description of the 43 published residency projects. The majority (n = 30; 68.2%) were published following a PGY2 residency. Table 2 also provides the overall publication rates among programs, as well as the publication rate of graduates per program. Of the 43 residency project publications, 30.2% were published by PGY1 graduates and 69.8% were published by PGY2 graduates. For publication rates of graduates by program, the overall PGY1 rate was 48.2% but varied among PGY1 subtypes, with 58.8% of PGY1 pharmacy graduates publishing their project. For PGY2 residents, the overall publication rate of graduates was 54.5%, but there was variability among the subtypes ranging from 25.0% to 87.5% of graduates (Table 2). The median (interquartile range, IQR) time to publication was 2.0 (1.5-2.6) years. The median (IQR) number of authors was 6 (5-6), with the resident serving as the first author on the majority of publications (n = 38; 88.4%). Twenty-eight projects (65.1%) were published in a pharmacy journal, with the remaining 15 (34.9%) published in a medical journal. Of the 37 publications with an impact factor available, the median was 1.9.

**Table 2.** Residency Research Projects That Were Published (n = 43)

Characteristic	Residency projects published (n = 43)
Residency type, No./No. total graduates (%)	
PGY1 residencies	
Overall	13/27 (48.2)
Community	3/9 (33.3)
Managed care	0/1 (0)
Pharmacy	10/17 (58.8)
PGY2 residencies	
Overall	30/55 (54.5)
PGY2 ambulatory care	14/21 (66.7)
Cardiology	3/6 (50.0)
HIV	3/8 (37.5)
Internal medicine	2/8 (25.0)
Oncology	1/4 (25.0)
Pediatrics	7/8 (87.5)
Number of authors on publication, median (IQR)	6 (5-6)
Author position of resident, No. (%)	
1	38 (88.4)
2	4 (9.3)
3	0 (0)
4	0 (0)
5	1 (2.3)
Journal type, No. (%)	
Medical	15 (34.9)
Pharmacy	28 (65.1)
Time to publication, median (IQR), years	1.9 (1.3-2.6)

Abbreviations: HIV, human immunodeficiency virus; IQR, interquartile range; PGY1, postgraduate year 1; PGY2, postgraduate year 2.

For all residents, subsequent publications were evaluated regardless of the publication status of the residency project. Fifty-four residents (74.0%) had at least one subsequent publication. The number of residents with subsequent publications varied by graduation year, ranging from 60.0% to 100.0%. There was no statistically significant difference in the number of residents with subsequent publications between those who did vs did not publish their research project (35 [79.6%] vs 28 [71.8%], respectively; *P* = 0.41). Table 3 provides a comparison of subsequent publications based on years after residency graduation between those who did vs did not publish their residency project. There were no statistically significant differences in the rates of subsequent publication. However, there was a significant difference in the median (IQR) number of subsequent publications between those who did vs did not publish their research project (4 [2-6] vs 2 [1-5, respectively; *P* = 0.04).

**Table 3.** Number of Residents With Subsequent Publications After Residency Completion Based on the Publication Status of Their Residency Project^a^

			Residents with subsequent publications by residency project status		
Graduation years included	Year after residency	Residents with subsequent publications	Residency project published	Residency project unpublished	*P* value
2011-2019	1	9/73 (12.3)	4/38 (10.5)	5/35 (14.3)	0.73
2011-2019	2	33/73 (45.2)	20/38 (52.6)	13/35 (37.1)	0.18
2011-2019	3	21/73 (28.8)	12/38 (31.6)	9/35 (25.7)	0.58
2011-2018	4	22/65 (33.9)	15/36 (41.7)	7/29 (24.1)	0.14
2011-2017	5	19/61 (31.2)	13/35 (37.1)	6/26 (23.1)	0.24
2011-2016	6	13/53 (24.5)	10/31 (32.3)	3/22 (13.6)	0.12
2011-2015	7	12/46 (26.1)	7/27 (25.9)	5/19 (26.3)	1.00
2011-2014	8	9/37 (24.3)	5/21 (23.8)	4/16 (25.0)	1.00
2011-2013	9	5/26 (19.2)	4/16 (25.0)	1/10 (10.0)	0.62
2011-2012	10	3/17 (17.7)	3/12 (25.0)	0/5 (0.0)	0.52
2011	11	2/9 (22.2)	2/7 (28.6)	0/2 (0.0)	1.00

^a^Data shown as the number of residents divided by the total number of residents or number of residents who published or did not publish their residency project (%).

When specifically evaluating the 38 residents who published their project, 76.9% had subsequent publications and 23.1% did not (Table 4). There was a significant difference in the first position after residency between the groups (*P* = 0.02). There was no statistically significant difference in the time to publish their research project, impact factor of the journal, or type of journal. However, there was a significant difference in the median (IQR) number of authors on the residency publication between residents who did vs did not have subsequent publications (6 [5-6] vs 4 [4-5)], respectively; *P* = 0.002). In addition, there was also a significantly higher median (IQR) coauthor H-index in 2021 for those who did vs did not have subsequent publications (15 [13-20] vs 10 [10-15], respectively; *P* = 0.02).

**Table 4.** Subanalysis of Published Residency Research Projects by Subsequent Publication Status

Variable	No subsequent publications	Subsequent publications	
Results by resident	n = 9	n = 29^a^	*P* value
First position after residency, No. (%)			0.02
Clinical pharmacist	9 (100.0)	13 (44.8)	
Academia	0 (0)	13 (44.8)	
Additional training	0 (0)	3 (10.3)	
Results by project	n = 9	n = 34	
Residency type, No. (%)			0.70
PGY1	2 (22.2)	11 (32.4)	
PGY2	7 (77.8)	23 (67.7)	
Time to publish residency research project, median (IQR), years	2.17 (1.59-2.59)	1.92 (1.34-2.51)	0.55
Time to publish residency research project (year categories), No. (%)			0.77
≤1	0 (0)	3 (8.8)	
1-2	4 (44.4)	15 (44.1)	
>2	5 (55.6)	16 (47.1)	
Journal type for residency project publication, No. (%)			1.0
Medicine	3 (33.3)	12 (35.3)	
Pharmacy	6 (66.7)	22 (64.7)	
Impact factor of journal for residency research project publication, median (IQR)	1.53 (1.15-2.43)^b^	1.57 (1.27-2.41)^c^	0.78
Number of authors on publication, median (IQR)	4 (4-5)	6 (5-6)	0.002
Author position of resident, No. (%)			0.23
1	7 (77.8)	31 (91.2)	
2	1 (11.1)	3 (8.8)	
3	0 (0)	0 (0)	
4	0 (0)	0 (0)	
5	1 (11.1)	0 (0)	
Highest coauthor H-index in 2021, median (IQR)	10 (10-15)	15 (13-20)	0.02

Abbreviations: IQR, interquartile range; PGY1, postgraduate year 1; PGY2, postgraduate year 2.

^a^Four residents published both their PGY1 and PGY2 projects.

^b^Impact factor available for 7 publications.

^c^Impact factor available for 29 publications.

To assess the time from residency graduation to publication, a multiple-variable Cox proportional regression analysis was performed. No difference in rate of publishing residency projects was found between PGY2 vs PGY1 residents (aHR, 1.25; 95% CI, 0.59-2.66; *P* = 0.56) and for residents who subsequently entered academia full time compared to those who became clinical pharmacists (aHR, 1.46; 95% CI, 0.67-3.19; *P* = 0.34). However, residents who completed additional training (ie, a research fellowship and/or graduate school) after residency had a 7.34 times faster time to publication than those who took a position as a clinical pharmacist (aHR, 7.34; 95% CI, 1.65-32.69; *P* = 0.01).

A ZIP regression analysis was used to determine variables associated with subsequent publications. This model was chosen owing to the excess of residents with no subsequent publications (27.4%) and because the variance was greater than the mean for the total number of subsequent publications. Using a 3-variable Poisson model, those who entered academia had an adjusted increase of 75% in the rate of subsequent publications (odds ratio [OR], 1.75; 95% CI, 1.28-2.40; *P* < 0.01) compared to those who accepted a clinical pharmacist position, while those who completed additional training following residencies had an adjusted increase of 431% in the rate of subsequent publications (OR, 4.31; 95% CI, 3.59-7.86; *P* < 0.01) compared to those who accepted a clinical pharmacist position. For each year following residency graduation, the adjusted rate of subsequent publications decreased by 13% (OR, 0.87; 95% CI, 0.82-0.93; *P* < 0.01).

## Discussion

In this study, we found that individualized research committees coupled with a structured research development program resulted in a research publication rate of 52.1%. This finding is difficult to compare to publication rates reported in the literature owing to significant variability from 2% to 86% in publication rates depending on the type of residency programs analyzed (ie, PGY1 only vs PGY1 and PGY2), population focus (eg, pediatrics), and support provided for the research projects.^8-11^ Our publication rate varied by specific program, with 87.5% of PGY2 pediatric residents publishing their project vs 25% of PGY2 internal medicine/oncology residents. Additionally, we noted that 54 residents (74.0%) had at least one subsequent publication after residency graduation, and residents who took an academic position or completed additional training after residency graduation (ie, a research fellowship and/or graduate school) had greater odds of a subsequent publication compared to those taking a clinical pharmacist position following graduation.

A variety of different approaches have been implemented to foster research skill development and increase research project publication. Several programs have instituted the use of a research team including preceptors with research experience and/or biostatical expertise to guide residents.^10,19,20^ Several of these provided oversight for all residents in their program (ranging from 26 to 31 residents) rather than creating individual committees for each individual resident.^19,20^ Olson and colleagues^10^ implemented an overall research committee for 36 PGY1 pharmacy residents, and they noted a significant increase in publication rate from 46.7% at baseline to 86.1% (*P* = 0.001). In contrast, our publication rate before implementation of the individualized research committee was anecdotally noted to be less than 25% for PGY1 and PGY2 residents. Additionally, our OUCOP residents had an individualized team that included a primary content mentor, their RPD, at least one additional content and/or practice expert, and an individual with biostatistical and study design expertise.

Addressing overall knowledge of research principles and skill development is another element of our research program. Recently, examples have been published of research development educational series in addition to a research team approach as part of a formal research certificate program.^21,22^ These programs utilized a nonindividualized research team approach but also implemented a longitudinal structured curriculum that covered several aspects of the research process. Darko and colleagues^21^ reported statistically significant improvements in a survey of their residents’ confidence to perform various research activities, including the publication process, as well as an assessment of their overall foundational knowledge of research (61.3% vs 84.7%; *P* = 0.002). However, the majority were PGY1 residents (12 PGY1 and 1 PGY2), and the sample size for the survey was low (n = 6). Weeda and colleagues^22^ found that, after implementing a research certificate program, there were improvements in confidence and attitudes toward research; however, there was no significant change in research knowledge from baseline. Although publication rates were not evaluated, these findings speak to the impact that a structured research development program may have on residents’ confidence and abilities. Although our research program does not offer a certificate, the topics covered in such programs are in line with those in our research program.

Another approach that programs have initiated is to institute protected time for research. Morbitzer and colleagues^23^ identified programmatic, project, and trainee barriers to completion of scholarship and research activities, and they noted that time constraints are major barriers for completing projects. Historically, many residency programs have allowed for a 1-month rotation in December and encouraged residents to complete their research following rotation responsibilities or in their spare time.^24^ However, given that many residents attend the ASHP Midyear Clinical Meeting and are given holiday leave in December, this may not allow concentrated time for research. Many have advised that quality research is best performed longitudinally with protected time during various intervals.^23-25^ All OUCOP residents had their research projects administered via a longitudinal learning experience where they were able to work on aspects of their research throughout the academic year. The residents were also given a required 1-month concentrated research rotation. Anecdotally, most OUCOP residents completed their research month in January through March.

Other approaches for enhancing a resident’s research experience include flipped research programs and multisite residency research programs. One benefit of the flipped research model (in which previous residents develop and submit an IRB protocol for incoming residents) is that it eliminates the barrier of waiting for IRB approval before initiating data collection. While this model has been shown to increase the likelihood of project completion and publication, there may be a number of downsides, including a potential lack of engagement of residents who may not have interest in the project.^23^ Shafeeq and colleagues^26^ noted that self-motivation to publish and mentor support following the PGY2 residency were independently associated with the publication success of PGY2 critical care residency projects.

Multisite projects involving residents at more than one institution may allow for projects with a larger sample size and for more robust research teams from participants at each site.^27^ Johnson and colleagues^27^ noted that this approach allows for increased mentorship of residents at programs that lack content mentors with research experience and biostatical expertise. As a surrogate marker for mentorship, we evaluated the H-index score for all coauthors on published projects. We found a significantly higher median H-index score for those who had subsequent publications vs those who did not. Additionally, there was as higher number of coauthors on published projects for those who had subsequent publications vs those who did not. These data indicate that both quality and quantity of research mentors may allow greater research exposure and development of research skills following residency graduation.

Of the 43 projects that were published, the majority were retrospective, published in a pharmacy journal, and published 2 years following residency graduation. These findings are similar to those in other published studies evaluating residency research projects.^8,10^ The majority of residents (88.4%) were the first author on their publication with a few serving as the second or fifth author. These findings seem to be in contrast to those of Olson and colleagues,^10^ who noted that only 29.0% of residents served as the first author and 6.5% were not listed as an author on their project publications. Determination of author order may differ by institution, and residents may be moved down in the author order if they were not as actively involved in pursuing publication.

We noted that 54 residents (74.0%) had at least one subsequent publication after residency graduation. However, there was no difference in subsequent publications among those who did and did not publish their project. These findings differ from those of Stranges and Vouri,^16^ who evaluated 152 residents who presented their projects at the Great Lakes Pharmacy Resident Conference. These authors noted that 36.2% had a subsequent publication; they found a difference in those who had at least one subsequent publication between those who did vs did not publish their projects (50% vs 22.4%; *P* < 0.001). It is likely that we did not find a significant difference because our overall rate of subsequent publication was higher. OUCOP encourages residents to coauthor an additional review article or case report, so some subsequent publications may have been initiated during residency.

As an additional analysis, the subsequent publication rate was analyzed on the basis of years after residency graduation. We noted no statistically significant differences in the number of subsequent publications by year after graduation. Miller and colleagues^8^ conducted a similar analysis in 434 PGY1 and PGY2 residents who completed a pediatric-focused research project over a 10-year period. They noted a significant difference in subsequent publications 2, 5, and 9 years after residency graduation. It is difficult to identify the factors that may influence subsequent publication following residency graduation. We analyzed the first position following residency and found that residents who took a position in academia and those completing additional training after graduation had a 75% and 431% increase, respectively, in the number of subsequent publications compared to those taking a clinical pharmacist position. Previous studies have demonstrated that residents who take academic positions are more likely to have subsequent publications and a greater number of publications.^8^ This is not surprising given that publications are often required for academia-based positions.

There were several limitations to this study. First, all our residency programs were based out of a college of pharmacy within an academic medical center. Thus, both faculty and nonfaculty preceptors have greater support for research activities and have a higher expectation for publication. Second, we did not survey or directly contact each resident to obtain data in this project. Instead, all data were obtained via MEDLINE, EMBASE, Google Scholar, and Scopus. It is possible that not all publications were identified in our literature searches due to changes in names of residents or difficulty finding publications that are not indexed in MEDLINE. However, we repeated our literature search on several different platforms including EMBASE, Google Scholar, and Scopus. Third, it is possible that some of the PGY1 and PGY2 residents may have initiated an additional publication (eg, case report, review) during their residency year in addition to their research project. Therefore, this may have influenced the number of unique subsequent publications that were initiated after residency graduation. Given the design of this study, we were unable to elucidate whether a subsequent publication was started during the residency year. Fourth, we were not able to identify all potential factors affecting subsequent publication, including position changes and internal motivation for publication.

## Conclusion

Following implementation of an individualized research committee alongside research skill development, the overall publication rate was 52.1%. The majority of published residency research projects were retrospective studies published in a pharmacy journal approximately 2 years following graduation. Residents who took an academic position or completed additional training after residency had higher odds of subsequent publications after residency graduation than those who took a clinical pharmacist position following graduation. Future studies should identify other strategies that may aid in enhancing the research skills of residents to increase publication rates and aid the transition to an independent research investigator.

## Supplementary Material

zxac233_suppl_Supplementary_Material
