# Supplementary material for: Publication rates of pharmacy residents involved in a team-based research program
Source: Am J Health Syst Pharm. 2022 Aug 18;79(23):2141–9. doi: 10.1093/ajhp/zxac233 (PMC10268584; doi:10.1093/ajhp/zxac233)
Supplement: zxac233_suppl_Supplementary_Material [file zxac233_suppl_supplementary_material.docx]

**eTable 1.** Overview of the Team-Based Research Program and Components of the Research Policy

| **Timeline** | **RRC Roles** | **RPD Roles** | **Mentor Roles** | **Committee Roles** | **Resident Roles** | **Educational Research Topics for Residents (Hours per activity)^a^** |
| --- | --- | --- | --- | --- | --- | --- |
| July |  | - Introduce resident to research mentor - Participate in committee meetings on research design | - Provide overview of project to resident - Assist resident in setting up & conducting committee meetings | - Participate in committee meetings on research design | - Meet with mentor to gain understanding of project - Perform literature search of the topic and refine the research question - Set up initial committee meeting & subsequent meetings | - CITI^b^ training module (4.5 hours) - OUHSC^c^ IRB^d^ additional CITI training module (0.5 hours) - Working with research team (0.5 hours) - Biostatistics refresher (1.5 hours) |
| August | - Review IRB protocols | - Participate in committee meetings on research design - Provide formative feedback on IRB protocol | - Participate in committee meetings on research design - Provide formative feedback on IRB protocol | - Participate in committee meetings on research design - Provide formative feedback on IRB protocol | - Conduct committee meetings & develop research plan - Develop IRB protocol | - Manuscript writing (1 hour) - Setting up spreadsheets for data analysis (2 hours) |
| September | - Review IRB protocols - Quarterly update on research progress | - Provide formative feedback on IRB protocol | - Provide formative feedback on IRB protocol - Quarterly evaluation | - Provide formative feedback on IRB protocol | - Make final revisions to IRB protocol - Submit protocol to RRC - Submit protocol to IRB | - Writing abstracts (1 hour) |
| October |  |  |  |  |  |  |
| November |  |  | - Train resident on data collection process |  | - Data collection (pending IRB approval) |  |
| December | - Quarterly update on research progress |  | - Train resident on data collection process - Quarterly evaluation |  | - Data collection - Prepare 1^st^ manuscript draft (title page, intro, & methods) |  |
| January |  | - Review 1^st^ manuscript draft/provide feedback | - Assist resident on data collection issues - Review 1^st^ manuscript draft/provide feedback | - Participate in discussion of any data collection issues (as needed) | - Data collection |  |
| February |  |  |  | - Participate in discussion of any data collection issues (as needed) | - Data collection |  |
| March | - Quarterly update on research progress |  | - Quarterly evaluation - Assist resident with interpretation of statistical analyses | - Selection of potential journal for publication - Discussion of study results (if available) | - Data collection - Data analysis (if data collection completed) | - Choosing a journal & overview of manuscript submission & review process (1 hour) |
| April | - Provide feedback on platform presentations | - Review 2^nd^ manuscript draft/provide feedback | - Assist resident with interpretation of statistical analyses - Review 2^nd^ manuscript draft/provide feedback | - Discussion of study results - Feedback on platform presentation | - Data analysis - Prepare 2^nd^ manuscript draft (preliminary results & table shells) - Practice platform presentation to research committee - Potential revisions to data collection and/or analysis based on committee feedback (if applicable) |  |
| May | - Review & update research policy | - Identify project for incoming resident & develop committees |  | - Continued discussion of study results (if applicable) | - Prepare results & discussion section of manuscript |  |
| June |  | - Review final manuscript - Finalize committee for incoming resident | - Review final manuscript - Complete final evaluation | - Review final manuscript - Accept committee appointment for incoming resident | - Submit final manuscript (June 20) |  |

Residents are encouraged to also read the ASHP Research Fundamental Series that may supplement the interactive sessions and cover additional topics.^16^; ^b^CITI = The Collaborative Institutional Training Initiative; ^c^OUHSC = University of Oklahoma Health Sciences Center; ^d^IRB = Institutional review board
